# Platelet-Rich Plasma (PRP)-Assisted Full-Thickness Skin Grafting for Tendon-Exposed Foot Wound in a Diabetic Patient: A Case Report

**DOI:** 10.7759/cureus.105758

**Published:** 2026-03-24

**Authors:** Actheinay Cruz Cobo, Yelka Matos Furones, Luis F Gonzalez Vazquez, Jose I Gamboa Arisso, Elizabeth Blanco Espinosa, Chloe M Asante

**Affiliations:** 1 Plastic and Reconstructive Surgery, Medicina Integra Specialty Clinic, Cancún, MEX; 2 North Georgia Clinical Research/Neurology, Alcanza Clinical Research, Woodstock, USA; 3 Anesthesiology, Geisinger Medical Center, Danville, USA; 4 General Medicine, Jimmy Hirtzel Hospital, Miami, USA; 5 General Practice, Ceda Orthopedic Group, Miami, USA; 6 General Surgery, Surgery Residency in Hospital Arnaldo Milian, Villa Clara, CUB

**Keywords:** activated platelet-rich plasma, full-thickness skin graft, reconstructive surgery, soft tissue coverage, tendon exposure

## Abstract

Traumatic foot wounds in patients with diabetes mellitus pose significant reconstructive challenges, especially when tendon exposure limits the feasibility of simple closure techniques and advanced microsurgical options are not available in low-resource settings. Platelet-rich plasma (PRP) is increasingly used as an adjunct to enhance wound bed quality by supporting tissue regeneration and vascularization, potentially improving the success of grafting procedures. We present the case of a 63-year-old man with type 2 diabetes mellitus who sustained a traumatic dorsal foot wound with exposed extensor tendons and was managed with serial wound care and three applications of activated PRP, resulting in progressive granulation tissue formation and improved vascularity. Definitive coverage was achieved using a manually harvested, fenestrated full-thickness skin graft (FTSG) from the medial aspect of the left arm in a resource-limited hospital setting, with primary closure of the donor site. A small central area of partial graft necrosis healed by secondary intention, and complete graft take was ultimately achieved. This case illustrates how PRP-assisted wound bed optimization can facilitate successful FTSG coverage of tendon-exposed traumatic defects in diabetic patients when more complex reconstructive options are unavailable, offering a practical solution in environments with limited resources.

## Introduction

Traumatic foot wounds in patients with diabetes mellitus represent a clinically relevant subset of lower extremity injuries. Although ulceration remains the most common cause of diabetic foot complications, trauma accounts for approximately 10-20% of foot wounds requiring surgical evaluation in hospital-based series worldwide [1]. In reconstructive practice, skin grafts constitute 40-60% of lower extremity soft tissue coverage procedures, whereas local, regional, or free flaps account for 20-40%, reflecting differences in defect complexity, vascular status, and institutional resources [2-5].

In high-income countries, such as the United States, traumatic foot injuries in individuals with diabetes are less frequent than ulcer-related defects, yet they still represent a meaningful proportion of limb salvage cases. National reconstructive databases show that skin grafting remains the most commonly performed soft tissue coverage technique for traumatic lower extremity wounds, while flap-based reconstruction is concentrated in specialized centers with microsurgical capability [6-8].

In contrast, many hospitals in low- and middle-income regions - including parts of Mexico - face significant limitations in access to microsurgical equipment, specialized personnel, and advanced reconstructive technologies. These constraints often necessitate the use of simpler, cost-effective techniques, even for complex wounds involving exposed tendon or bone [9].

Platelet‑rich plasma (PRP) has gained attention as an adjunct capable of enhancing angiogenesis, fibroblast proliferation, and extracellular matrix deposition, thereby improving wound bed quality in complex wounds [7-10]. Although most evidence describes its use in chronic or diabetic wounds [8-10], its role in preparing acute traumatic tendon-exposed defects for grafting is less well documented.

This report describes the successful use of PRP-assisted wound bed optimization followed by a fenestrated full-thickness skin graft (FTSG) in a diabetic patient with a traumatic dorsal foot wound and tendon exposure, treated in a resource-limited hospital setting in Mexico.

## Case presentation

**Figure 1:**
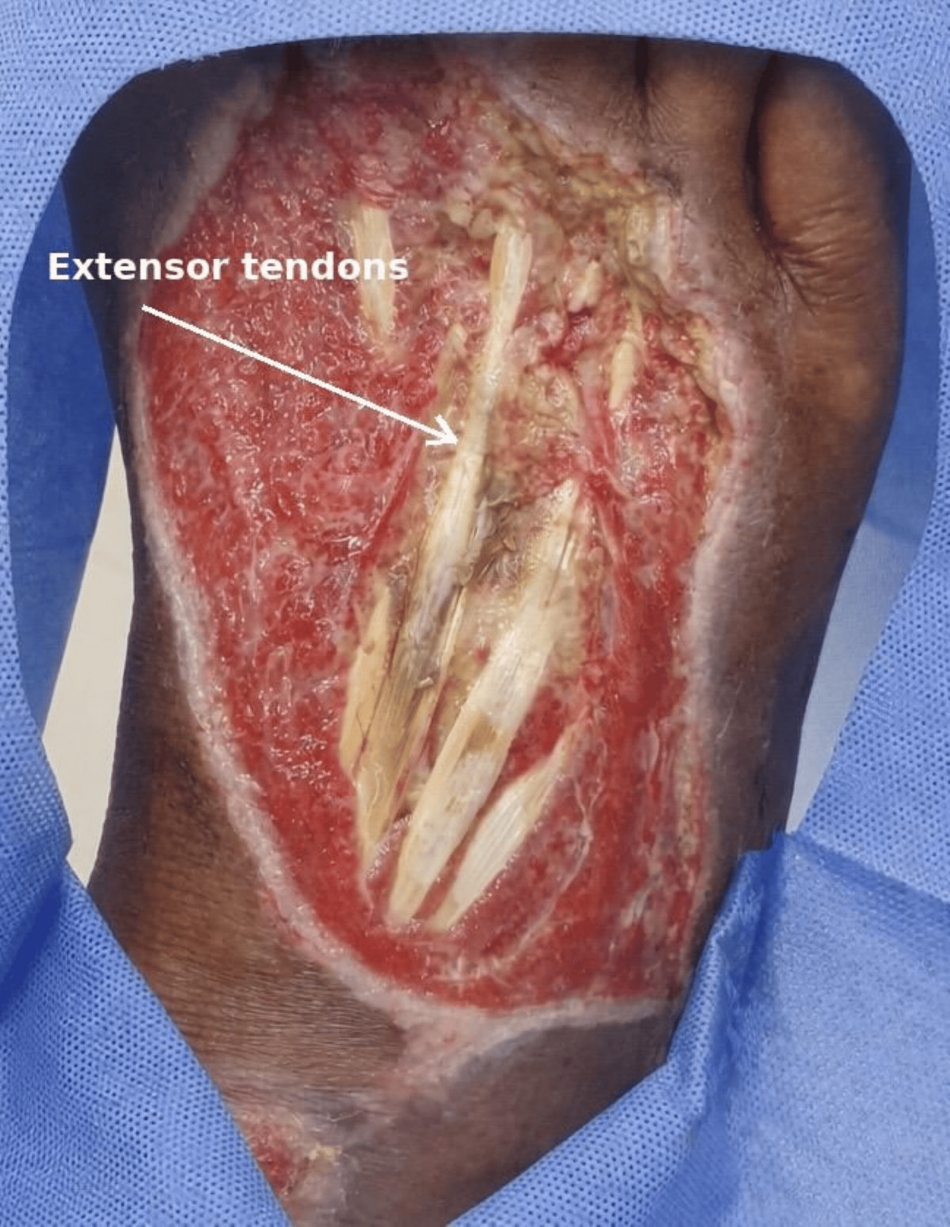
Dorsal foot wound showing an 8 × 5 cm full‑thickness defect with exposure of the extensor tendons.

A 63-year-old male with a history of diabetes mellitus sustained a traumatic injury to the dorsum of the right foot following a motor vehicle accident. Clinical examination revealed an approximately 8 × 5 cm full-thickness skin defect with loss of soft tissue coverage and exposure of the tendons of the extensor apparatus (Figure 1). The wound bed demonstrated limited granulation tissue formation, although no areas of active necrosis or bone exposure were identified. There were no motor, sensory, or reflex disorders on physical examination. Peripheral pulses were palpable, and no clinical signs of peripheral arterial disease were identified.

**Figure 2:**
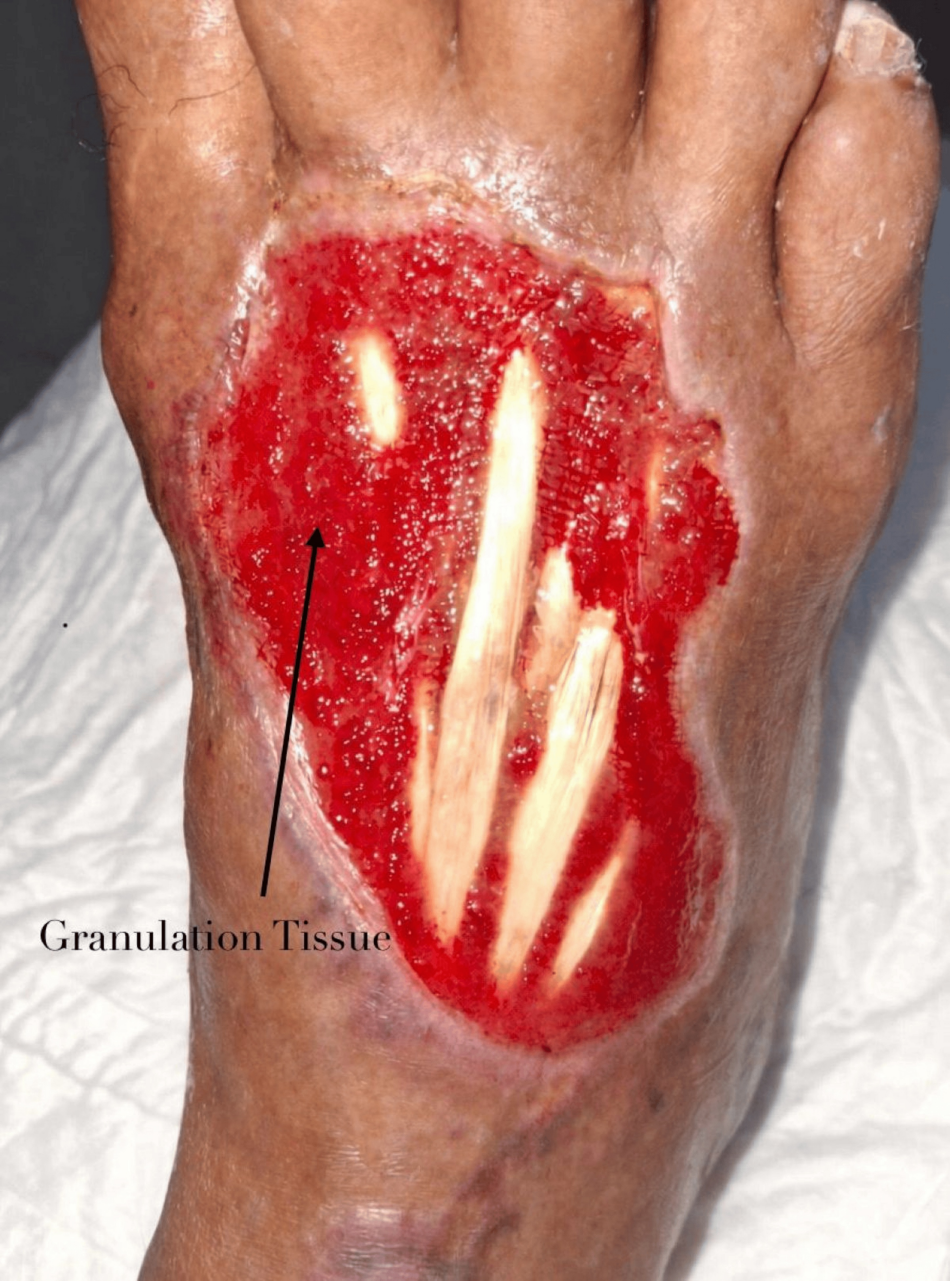
Granulation tissue formation after three sessions of activated platelet-rich plasma applied to the wound bed.

Initial management consisted of conventional wound care, including antiseptic cleansing with povidone-iodine and Microdacyn®, followed by topical application of 1% silver sulfadiazine. Despite these measures, the wound demonstrated minimal progression toward granulation. Given the stagnant evolution, wound bed optimization was initiated using activated PRP. The preparation was activated with calcium chloride at a 1:10 ratio, and a total of 2 mL was infiltrated into the perilesional tissue and wound bed in three sessions performed on alternate days prior to grafting. A marked improvement in granulation was observed only after the initiation of PRP therapy (Figure 2).

Negative pressure wound therapy (NPWT) promotes granulation through controlled sub-atmospheric pressure that reduces edema, enhances perfusion, and stimulates cellular proliferation within the wound bed [8]. In contrast, PRP delivers concentrated growth factors that accelerate angiogenesis, fibroblast activity, and extracellular matrix deposition through direct biochemical stimulation. While NPWT provides mechanical and environmental advantages that are particularly effective in large or exudative wounds, PRP enhances the biological healing cascade and may be especially beneficial in smaller defects or in patients with impaired healing potential. In this case, PRP was selected based on the plastic surgeon’s assessment that a biologically targeted approach would be more suitable than NPWT for a patient with diabetes mellitus.

Preoperative laboratory evaluation included a complete blood count, fasting glucose, serum creatinine, and a full coagulation profile. All values were within normal range (NR) for adult males in our region (Table 1).

**Table 1:** Preoperative laboratory evaluation.

Parameter	Result	Reference Range (Adult Male)	Units
Hemoglobin	14.2	13.5-17.5	g/dL
Leukocytes (WBC)	9	4-11	K/µL
Platelets	400	150-450	K/µL
Fasting glucose	88	70-99	mg/dL
Creatinine	0.9	0.7-1.3	mg/dL
Prothrombin time (PT)	12	11-14	seconds
INR	0.9	0.8-1.2	-
Activated partial thromboplastin time (aPTT)	30	25-35	seconds
Fibrinogen	250	200-400	mg/dL
Wound secretion culture	No pathogenic growth	-	-

Surgical technique

Anesthetic Considerations

The skin was prepared with 2% chlorhexidine solution. Local infiltrative anesthesia with 1% lidocaine combined with epinephrine 1:100,000 was administered at the donor site to enhance hemostasis and improve operative visibility. The recipient site was anesthetized using perilesional infiltration with 1% lidocaine without vasoconstrictors to avoid compromising perfusion in a diabetic lower-extremity wound.

Graft Harvesting

**Figure 3:**
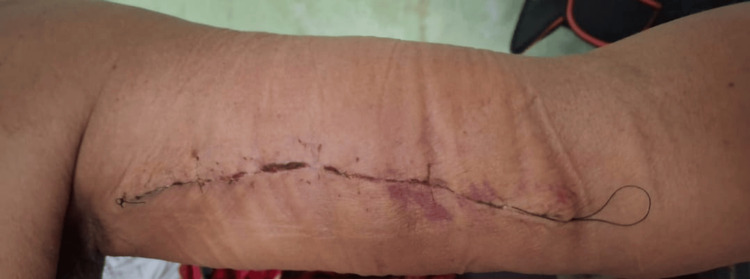
Donor site on the medial left arm following primary closure.

**Figure 4:**
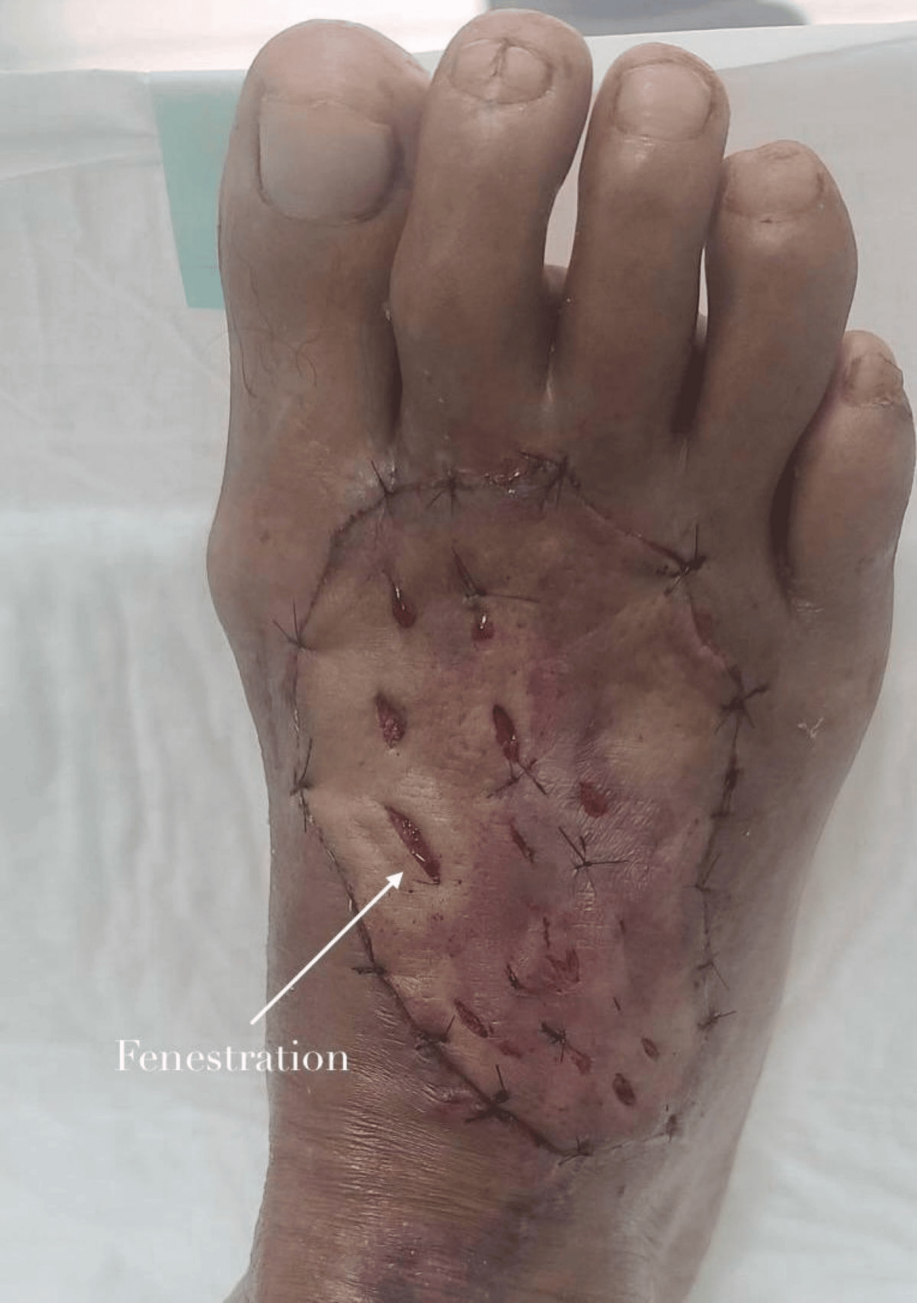
Full-thickness skin graft harvested from the medial left arm after defatting and fenestration.

A dermatome was not available in the operating room, and therefore, the graft was harvested manually using a scalpel. The medial aspect of the left arm was selected as the donor site due to sufficient skin laxity, allowing primary closure without tension (Figure 3). An FTSG was harvested manually using a scalpel, meticulously defatted, fenestrated, and stored in saline until implantation (Figure 4).

Preparation of the Recipient Bed

The wound bed was debrided until a clean, well-vascularized surface was achieved.

Graft Placement and Fixation

The FTSG was positioned over the defect, secured with absorbable sutures, and covered with nitrofurazone-impregnated mesh, a moist saline compress, and a Brown tie-over dressing.

Postoperative Course

**Figure 5:**
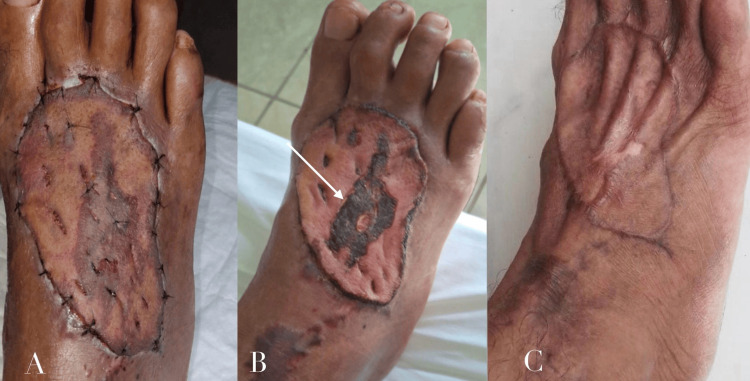
A) Postoperative appearance at five days showing a small central area of partial graft necrosis. B) Postoperative appearance at fifteen days demonstrating persistent but limited central graft necrosis (white arrow) with ongoing secondary‑intention healing. C) Healed graft site with complete graft take.

A small central area of partial graft necrosis developed, which healed by secondary intention without compromising overall graft survival. The donor site healed by primary intention. The patient recovered uneventfully and regained functional use of the foot during early follow-up (Figure 5).

Ethical approval

According to institutional regulations, formal ethics committee approval was not required for this single-patient case report. The patient provided written informed consent for publication of anonymized clinical information and images.

## Discussion

Traumatic dorsal foot wounds with tendon exposure in patients with diabetes present a complex reconstructive challenge due to impaired perfusion, neuropathy, and increased susceptibility to infection [6]. FTSGs are traditionally considered less reliable in this context because of their high metabolic demand and limited tolerance for poorly vascularized beds. For this reason, flap-based reconstruction is often preferred for its superior vascularity and durability [10-14]. However, flap success depends heavily on the patient’s vascular status, comorbidities, and the availability of specialized microsurgical resources-factors that frequently limit their feasibility in low-resource environments [11,15].

Although the reconstructive ladder favors simpler techniques before more complex options, tendon-exposed defects remain particularly challenging. Loss of the paratenon significantly reduces local vascularity [16]. Advanced reconstructive modalities - including dermal regeneration templates, microvascular free flaps, and robotic or anastomosis-based microsurgery - can achieve excellent outcomes but require equipment, expertise, and financial resources not universally available [1,11-18]. Local and regional flaps may offer reliable coverage, yet their applicability in the foot is limited by restricted soft-tissue mobility and the risk of pedicle compromise [9,10].

In this setting, PRP-assisted wound bed preparation followed by a fenestrated FTSG provided a practical and cost-effective alternative. PRP enhances angiogenesis, fibroblast proliferation, and extracellular matrix deposition, improving the biological quality of the wound bed and facilitating graft adherence even in compromised environments [2-5,7]. Although most evidence describes its use in chronic wounds and diabetic foot ulcers [3,5,7], this case involved an acute traumatic wound in a patient with diabetes. PRP application resulted in a well-vascularized granulation surface suitable for graft placement, despite the absence of an intact paratenon.

The favorable outcome challenges the prevailing assumption that FTSGs are unreliable for tendon-exposed defects in diabetic lower extremities [4,17]. It demonstrates that, when the wound bed is adequately optimized and advanced reconstructive options are unavailable, FTSGs can achieve reliable and functionally meaningful coverage. This case underscores the importance of tailoring reconstructive strategies to the patient’s clinical, socioeconomic, and infrastructural context and supports reconsidering FTSGs as a viable option in selected complex foot wounds, particularly in low-resource settings where flap surgery may not be feasible [1,15,16].

Limitations

This report describes the outcome of a single patient, which inherently limits the generalizability of the findings. The favorable result cannot establish the reproducibility or comparative effectiveness of PRP-assisted wound bed preparation or fenestrated full-thickness skin grafting in similar clinical scenarios. No objective perfusion measurements, histologic assessments, or long-term functional evaluations were performed, which restricts the ability to draw broader conclusions regarding durability or long-term graft behavior. Further studies involving larger cohorts and controlled comparisons are needed to clarify the role of PRP-optimized FTSGs in tendon-exposed wounds, particularly in patients with diabetes.

## Conclusions

The combination of PRP-assisted wound bed preparation and a fenestrated FTSG achieved successful coverage of a traumatic dorsal foot defect with tendon exposure in a patient with diabetes. Although conclusions cannot be generalized from a single case, this experience suggests that, when the wound bed is carefully optimized and advanced reconstructive options are unavailable, FTSGs may represent a reasonable and context-appropriate option for selected lower-extremity wounds. This case highlights the importance of adaptable, resource-conscious strategies in settings where microsurgical or flap-based reconstruction is limited or impractical.
